# Supplementary material for: Early detection of cardiotoxicity in children receiving adriamycin: A comparison of tissue Doppler and standard echocardiography with Troponin I as an early marker
Source: PLoS One. 2025 Aug 7;20(8):e0328632. doi: 10.1371/journal.pone.0328632 (PMC12331081; doi:10.1371/journal.pone.0328632)
Supplement: Attachment — Submitted filename: Review cardiotoxicity.docx [file pone.0328632.s001.docx]

**Review – Early Detection of Cardiotoxicity in Children Receiving Adriamycin: A Comparison of Tissue Doppler and Standard Echocardiography with Troponin I as an Early Marker**

I have read with great interest the paper "Early Detection of Cardiotoxicity in Children Receiving Adriamycin: A Comparison of Tissue Doppler and Standard Echocardiography with Troponin I as an Early Marker" by Farshidgohar et al. The manuscript explores the utility of echocardiographic methods, specifically tissue Doppler imaging (TDI), for the early identification of cardiac toxicity. Below are specific comments intended to enhance the clarity and depth of the manuscript.

In general, the manuscript would greatly benefit from a more comprehensive review of existing literature regarding the use of TDI and other echocardiographic measures to detect cardiotoxicity during oncology treatment (see suggested references below). Additionally, the authors should provide more detailed descriptions regarding the clinical course of the patients, specifically highlighting how abnormal TDI findings impacted clinical management. The manuscript would also be strengthened by including figures illustrating the echocardiographic changes due to treatment.

Methods

- The sentences: "The study included 50 pediatric patients diagnosed with cancer (leukemia, lymphoma, and solid tumors) who were undergoing Adriamycin treatment," and "The median cumulative dose of Adriamycin administered was 400 mg/m²," belong in the Results section. The Methods section should solely describe the study procedures.
- Please expand upon the inclusion criteria: Were all pediatric cancer patients (with leukemia, lymphoma, or solid tumors) approached, or were there additional selection criteria?

Results

- The "Echocardiographic Findings" section should be converted into paragraph form with complete sentences to align better with the style of an academic paper.
- For the statements: "Significant reductions in S’ and E’ velocities (p<0.001)," and "Right ventricular (RV) TAPSE showed a significant decline," please include specific numerical values.
- The sentence: "No significant changes in LVEF were observed one and six months post-treatment (mean LVEF: 64%, 63%, 62%, respectively)" requires the addition of 95% confidence intervals (CIs) for these means if the data distribution was indeed normal. If not normally distributed, please convert to median and IQR.
- In the sentence "In Tissue Doppler echocardiography, a significant decrease in the systolic function of the left ventricle was observed in all patients.” Please be more specific and provide actual results and Chi-square test results.
- The sentence “Additionally, we analyzed the frequency (%) of categorical variables to compare them across three stages using the Chi-square test (X²) (Table 2) belongs in the Methods section, whereas here you should present the findings derived from these analyses in the results.

Additional clarifications requested:

- Describe the clinical management of the patients included in the study, with focus on patients with abnormal echo / troponin values. Was Adriamycin discontinued for any patient? Did you initiate cardioprotective therapy (ACE inhibitors or beta-blockers)? Provide more clinical details, particularly for the two patients with elevated Troponin I levels: Did they continue treatment?
- Expand the first paragraph of the Results to include cohort demographics, detailed oncologic diagnoses, specific treatment durations, Adriamycin dosage, and follow-up durations.
- Were additional follow-up diagnostic tests (such as cardiac MRI or stress testing) conducted for any patients, especially those with abnormal echo results?
- Report any abnormal ECG findings (e.g., sinus tachycardia, decreased voltage, QT prolongation).
- Include a figure and/or video (can be in the supplementary material) demonstrating echocardiographic changes before and after treatment.
- Provide definitions for all abbreviations used in the manuscript (e.g., "SM").

Discussion

- Introduce a dedicated "Limitations" paragraph, addressing issues such as the lack of additional diagnostic cardiac tests, limitations and potential inaccuracies associated with the echocardiographic methods used, and absence of long-term follow-up data. In this paragraph you can also include the limitation of having troponin only immediately after the start of treatment.
- Incorporate references to other relevant studies, discussing how your findings relate to previous research and highlighting the novelty and added value of your work compared to existing evidence. Suggested references include:

<https://pmc.ncbi.nlm.nih.gov/articles/PMC7048119/>

<https://pubmed.ncbi.nlm.nih.gov/25577226/>

<https://pubmed.ncbi.nlm.nih.gov/22825896/>

<https://journals.lww.com/anti-cancerdrugs/abstract/2011/06000/chemotherapy_induced_cardiotoxicity__role_of_the.12.aspx>

<https://onlinelibrary.wiley.com/doi/10.5402/2012/870549>

<https://bjcardio.co.uk/2016/04/strain-imaging-and-anthracycline-cardiotoxicity/>
