# Supplementary material for: Early detection of cardiotoxicity in children receiving adriamycin: A comparison of tissue Doppler and standard echocardiography with Troponin I as an early marker
Source: PLoS One. 2025 Aug 7;20(8):e0328632. doi: 10.1371/journal.pone.0328632 (PMC12331081; doi:10.1371/journal.pone.0328632)
Supplement: Attachment — Submitted filename: Response to Reviewers.docx [file pone.0328632.s002.docx]

**Response to Review Comments**

**Submission ID PONE-D-25-05750; " Early Detection of Cardiotoxicity in Children Receiving Adriamycin: A Comparison of Tissue Doppler and Standard Echocardiography with Troponin I as an Early Marker"**

**Journal: Plos One**

I am grateful to the reviewers for their deep and thorough review. I have revised my current research paper based on their valuable suggestions and comments. I trust that my revisions have enhanced the paper to meet their expectations. Here are the specific responses to their comments, suggestions, and queries.

**Response to Reviewer**

**Reviewer 1**

 I have read with great interest the paper "Early Detection of Cardiotoxicity in Children Receiving Adriamycin: A Comparison of Tissue Doppler and Standard Echocardiography with Troponin I as an Early Marker" by Farshidgohar et al. The manuscript explores the utility of echocardiographic methods, specifically tissue Doppler imaging (TDI), for the early identification of cardiac toxicity. Below are specific comments intended to enhance the clarity and depth of the manuscript.

In general, the manuscript would greatly benefit from a more comprehensive review of existing literature regarding the use of TDI and other echocardiographic measures to detect cardiotoxicity during oncology treatment (see suggested references below). Additionally, the authors should provide more detailed descriptions regarding the clinical course of the patients, specifically highlighting how abnormal TDI findings impacted clinical management.

**Response. Thank you for your valuable feedback. We provided more detailed descriptions regarding the clinical course of the patients, specifically highlighting how abnormal TDI findings impacted clinical management in Result section.**

The manuscript would also be strengthened by including figures illustrating the echocardiographic changes due to treatment.

**Response. Thank you for your valuable feedback. Figure related to TAPSE added to the manuscript (Figure 2).**
Methods
• The sentences: "The study included 50 pediatric patients diagnosed with cancer (leukemia, lymphoma, and solid tumors) who were undergoing Adriamycin treatment," and "The median cumulative dose of Adriamycin administered was 400 mg/m²," belong in the Results section. The Methods section should solely describe the study procedures.

**Response. According to reviewer comment, demographic information of patients and dose of Adriamycin administered was transferred to result section.**
• Please expand upon the inclusion criteria: Were all pediatric cancer patients (with leukemia, lymphoma, or solid tumors) approached, or were there additional selection criteria?

**Response. Thank you for your valuable feedback. Inclusion criteria was expanded in method (Study Design).**
Results
• The "Echocardiographic Findings" section should be converted into paragraph form with complete sentences to align better with the style of an academic paper.

**Response. According to reviewer comment,** **the "Echocardiographic Findings" section was converted into paragraph form with complete sentences.**
• For the statements: "Significant reductions in S’ and E’ velocities (p<0.001)," and "Right ventricular (RV) TAPSE showed a significant decline," please include specific numerical values.

**Response. Thank you for your valuable feedback.** **We included specific numerical values.**
• The sentence: "No significant changes in LVEF were observed one and six months post-treatment (mean LVEF: 64%, 63%, 62%, respectively)" requires the addition of 95% confidence intervals (CIs) for these means if the data distribution was indeed normal. If not normally distributed, please convert to median and IQR.

**Response. According to reviewer comment, related findings were converted to median and IQR in Table 1. Results showed that between before, one, and six months’ post-treatment was significant based on nonparametric test.**
• In the sentence "In Tissue Doppler echocardiography, a significant decrease in the systolic function of the left ventricle was observed in all patients.” Please be more specific and provide actual results and Chi-square test results.

**Response. So thankful for your comment. The findings of Tissue Doppler echocardiography were expanded more specific and Chi-square test results presented.**

• The sentence “Additionally, we analyzed the frequency (%) of categorical variables to compare them across three stages using the Chi-square test (X²) (Table 2) belongs in the Methods section, whereas here you should present the findings derived from these analyses in the results.

**Response. In light of your insightful feedback, related sentence was presented in result section.**

Additional clarifications requested:
• Describe the clinical management of the patients included in the study, with focus on patients with abnormal echo / troponin values. Was Adriamycin discontinued for any patient? Did you initiate cardioprotective therapy (ACE inhibitors or beta-blockers)? Provide more clinical details, particularly for the two patients with elevated Troponin I levels: Did they continue treatment?

**Response. In light of your insightful feedback, we described the clinical management of the patients included in the study in result section (Troponin I Levels).**

• Expand the first paragraph of the Results to include cohort demographics, detailed oncologic diagnoses, specific treatment durations, Adriamycin dosage, and follow-up durations.

**Response. So thankful for your comment. According to reviewer comment, in the first paragraph of the Results added cohort demographics, detailed oncologic diagnoses, specific treatment durations, Adriamycin dosage, and follow-up durations.**
• Were additional follow-up diagnostic tests (such as cardiac MRI or stress testing) conducted for any patients, especially those with abnormal echo results?

**Response. It was not possible to perform the cardiac MRI for the patients in our study.**

• Report any abnormal ECG findings (e.g., sinus tachycardia, decreased voltage, QT prolongation).

**Response. According to reviewer comment, we reported ECG findings in Result section (Electrocardiographic Findings).**
• **Include a figure and/or video (can be in the supplementary material) demonstrating echocardiographic changes before and after treatment.**

**Response. Thank you for your valuable feedback. Figure related to TAPSE (Figure 2) was added to the manuscript.**
• Provide definitions for all abbreviations used in the manuscript (e.g., "SM").

**Response. In light of your insightful feedback, we added definitions for all abbreviations used in the manuscript at the end of manuscript.**

Discussion
• Introduce a dedicated "Limitations" paragraph, addressing issues such as the lack of additional diagnostic cardiac tests, limitations and potential inaccuracies associated with the echocardiographic methods used, and absence of long-term follow-up data. In this paragraph you can also include the limitation of having troponin only immediately after the start of treatment.

**Response. According to reviewer comment, we added Limitations of the study before conclusion section.**

• Incorporate references to other relevant studies, discussing how your findings relate to previous research and highlighting the novelty and added value of your work compared to existing evidence. Suggested references include:

**Response. According to reviewer comment, we referred to other relevant studies in Discussion section. In addition, we added novelty and value of our work compared to existing evidence (before Limitation section).**
