# Supplementary material for: Early detection of cardiotoxicity in children receiving adriamycin: A comparison of tissue Doppler and standard echocardiography with Troponin I as an early marker
Source: PLoS One. 2025 Aug 7;20(8):e0328632. doi: 10.1371/journal.pone.0328632 (PMC12331081; doi:10.1371/journal.pone.0328632)
Supplement: Attachment — Submitted filename: Review cardiotoxicity - RR1.docx [file pone.0328632.s003.docx]

**Review (RR1) – Early Detection of Cardiotoxicity in Children Receiving Adriamycin: A Comparison of Tissue Doppler and Standard Echocardiography with Troponin I as an Early Marker**

I thank the authors for addressing the previous comments and making notable improvements to the manuscript. However, there remain some important areas needing further clarification and revision to enhance the manuscript's overall quality. Specific points are detailed below.

**Introduction**

No comments

**Methods**

- “*Diastolic Function: Normal diastolic function was defined as an E/A ratio of 0.9–1.5. Abnormal diastolic function was considered if the E/A ratio was <0.9 or >2*.” Clarify how values between 1.5 and 2 were categorized, as the provided ranges do not currently cover these values. Did you intend the normal range to be 0.9–2?
- All echocardiographic variables you reference (LVEF, TAPSE, S’, E/E’, E/A) vary significantly by age across the 2–14 year range in your study. Applying adult-reference ranges universally is a substantial limitation and should be acknowledged explicitly in the discussion. You may, however, also mention that since comparisons were intra-patient (patients compared to themselves), then this limitation likely does not significantly impact your main results.

**Results**

- “The median follow-up duration for cardiac evaluation was six months.” Given that all patients had a minimum follow-up of 6 months, stating the median as exactly 6 months is unclear or possibly incorrect. Consider revising to: "All patients were followed for the 6-month duration of the study, and no further follow-up data were available," or clearly state the actual median and IQR for available follow-up durations.
- “Standard Doppler Echocardiography:” Please correct the formatting to properly align the section title. (remove the bullet-point format and align the paragraph with the rest of the text).
- The presentation of the statistical analysis results is currently difficult to follow. Clarify precisely what EF1, EF2, and EF3 represent. Consider revising to: "The Kolmogorov–Smirnov test indicated significant deviation from a normal distribution (statistic > 0.153, p < 0.05). The median EF at baseline, one month, and six months post-treatment were 65% (range: 50–78%), 65% (range: 50–75%), and 62% (range: 50–76%), respectively."
- “The mean S’ velocity before starting Adriamycin treatment, one month after treatment, and six months after treatment was 10.3 cm/s, 8.3 cm/s, and 8.3 cm/s,” and similarly, “The mean E’ velocity before starting Adriamycin treatment, one month and six months after treatment was 16.8 cm/s, 14 cm/s and 14 cm/s (p<0.001).” Confirm whether these values were normally distributed. If normally distributed, please include the 95% confidence intervals. If not, replace means with medians and interquartile ranges (IQR) for consistency and statistical appropriateness.

Please fix the formatting of Table 1 & 2 so they will be more readable (see screenshots). I believe it would be much easier to read Table 1 if it was formatted in the following way:

|  | Baseline | 1-Month | 6-Months | p-value |
| --- | --- | --- | --- | --- |
| EF | Median (IQR) | Median (IQR) | Median (IQR) |  |
| SM | … | … | … |  |
| E’ | … | … | … |  |
| E/A | … | … | … |  |
| …. |  |  |  |  |

And Table 2 if it was formatted in the following manner:

|  | Baseline | 1-Month | 6-Months | p-value |
| --- | --- | --- | --- | --- |
| E’* |  |  |  |  |
| ≥ 8 cm/s | Median (IQR) | Median (IQR) | Median (IQR) |  |
| < 8 cm/s | … | … | … |  |
|  | … | … | … |  |
|  |  |  |  |  |

Figures

- **Figure 1**: Create a figure comparing baseline measurements with measurements taken at either 1 or 6 months post-treatment. The current figure is too generic and less informative with respect to the study’s objectives. Consider including a visual comparison demonstrating echocardiographic doppler changes before and after treatment (as also suggested in the previous manuscript review).
- **Figure 2**: Adjust the y-axis to start at ‘y=0’. The current axis range artificially exaggerates the perceived effect.

**Discussion**

The discussion requires significant editing to eliminate repetition and redundancy. Revise this section to clearly and concisely include:

- One paragraph summarizing your key findings.
- One paragraph discussing relevant literature.
- One paragraph highlighting the strengths of this study compared to prior research.
- One paragraph clearly outlining limitations.
- A brief summary or conclusion statement.

The discussion section should be much shorter than its current form.

Ensure consistent citation formatting, using brackets ([]) rather than parentheses (()). For example, revise: “In alignment with these findings, our study observed early diastolic dysfunction in 80% of patients at one-month post-treatment (16),” to “In alignment with these findings, our study observed early diastolic dysfunction in 80% of patients at one-month post-treatment [16].”
