# Supplementary material for: Early detection of cardiotoxicity in children receiving adriamycin: A comparison of tissue Doppler and standard echocardiography with Troponin I as an early marker
Source: PLoS One. 2025 Aug 7;20(8):e0328632. doi: 10.1371/journal.pone.0328632 (PMC12331081; doi:10.1371/journal.pone.0328632)
Supplement: Attachment — Submitted filename: point-by-point response .docx [file pone.0328632.s005.docx]

Dear editors and reviewers,
Thank you very much for sending us the reviewers’ comments on our manuscript entitled “***Early Detection of Cardiotoxicity in Children Receiving Adriamycin: A Comparison of Tissue Doppler and Standard Echocardiography with Troponin I as an Early Marker”***. We appreciate the time and effort that the editors and the reviewers have dedicated to providing the valuable feedback on our manuscript. We are grateful to the reviewers for their insightful comments. We have incorporated changes to reflect the suggestions provided by the reviewers and improved the logic and the language of the manuscript. The revised contents are highlighted in blue within the revised manuscript.
Our point-by-point responses to the reviewers’ comments are as follows:

**Additional Editor Comments :**

Very good progress has been made in last two rounds of review, before I accept your manuscript for publication please make the following minor adjustments:
1) Please restate this paragraph in more informal way since these numbers can be found in Table 1:
"After testing the normality distribution of data,
Kolmogorov–Smirnov statistic more than 0.153 with a p-value <0.05 indicated significant
deviation from normality at α = 0.05: EF1 of baseline; median= 65 (50, 78), IQR= 10, EF2 of
after 1 month; median= 65 (50, 75), IQR= 8.25, and EF of after 6 months; median= 62 (50, 76),
IQR= 7.5. Therefore, there was significant changes in LVEF one and six months post-treatment
by Friedman test p<0.001."
2) When giving the IQR of certain variables (e.g. IQR) please give it in format with 25th and 75th percentile, not as single number since IQR can be asymmetric and that is not captured well by a single number.

Authors’ answer: Thank you very much for your suggestion. We rewrite this sentence and table1.

*“Due to the data not adhering to a normal distribution (Kolmogorov–Smirnov test, p < 0.05), LVEF values were presented as medians and interquartile ranges. Table 1 illustrates LVEF slightly declined over time, and the Friedman test confirmed a significant change at one and six months after Adriamycin treatment (p < 0.001).”*

Reviewer #1: Thank you for addressing all my comments. Two small things that should be changed before publications:
1. I would remove the text you added "A 95% confidence interval was used for the standard normal distribution. Whereas, data that do not follow a normal distribution was presented as medians and interquartile ranges (IQR) (Table 1)." -- this should be in methods and not results. In the results I only meant that when you mention numbers in the text, you should include in parenthesis the 95% CI. You don't need to describe the method used here, just include the resulting numbers.

Authors’ answer: Thank you very much for drawing attention to this. We fixed it.

2. Work with the editorial office on the formatting of the tables. For example, change 'befor' in table 1 to 'before', and either remove all the asterisks there or include the description of each an each sequence of asterisks (e.g. what does *, **, *** etc. mean..).

Authors’ answer: Thank you very much for your suggestion. We fixed it.
